# A Moving Target: Studying the Effect of Continuous Transcutaneous CO_2_ Monitoring in ELBW Infants During an Equipoise Shift

**DOI:** 10.3390/jcm13216472

**Published:** 2024-10-29

**Authors:** Liron Borenstein-Levin, Noa Avishay, Ori Hochwald, Orit Soffer, Shmuel Arnon, Arieh Riskin, Ayala Gover, Karen Lavie-Nevo, Alon Haham, Justin Richardson, Ilya Rozin, Amir Kugelman

**Affiliations:** 1 Department of Neonatology, Rambam Health Care Campus, Haifa 3525408, Israel; ori.hochwald@gmail.com (O.H.); oritsof@gmail.com (O.S.); amirkug@gmail.com (A.K.); 2 Rappaport Faculty of Medicine, Technion-Israel Institute of Technology, Haifa 3109601, Israel; noa.avishai@gmail.com (N.A.); arik.riskin@gmail.com (A.R.); ayalagover@gmail.com (A.G.); klavie@gmail.com (K.L.-N.); 3 Department of Neonatology, Meir Medical Center, Kfar-Saba 4428163, Israel; shmuelar@clalit.org.il; 4 Sackler School of Medicine, Faculty of Medical and Health Sciences, Tel Aviv University, Tel Aviv 6927846, Israel; 5 Department of Neonatology, Bnai Zion Medical Center, Haifa 3339419, Israel; 6 Department of Neonatology, Carmel Medical Center, Haifa 3436212, Israel; 7 Department of Neonatology, Tel Aviv Sourasky Medical Center, Tel Aviv 6423906, Israel; alonhaham@yahoo.com; 8 Department of Neonatology, Soroka Medical Center, Be’er Sheva 84101, Israel; justinri@clalit.org.il; 9 Department of Neonatology, Kaplan Medical Center, Rehovot 7661041, Israel; ilyaro@gmail.com

**Keywords:** non-invasive CO_2_ monitoring, premature infant, transcutaneous CO_2_ monitoring, intraventricular hemorrhage

## Abstract

**Objectives**: To assess whether continuous non-invasive pCO_2_ monitoring by transcutaneous pCO_2_ monitor (TCpCO_2_) among extremely low birth weight (ELBW) premature infants, during the first week of life, will decrease the rate of high-grade intraventricular hemorrhage (IVH) or periventricular leukomalacia (PVL) or the combined outcome of IVH/PVL and death. **Methods**: This was a prospective, observational, multicenter study. Due to ethical constraints, allocation was based on TCpCO_2_ monitor availability. ELBW infants were either monitored by TCpCO_2_ monitor (Sentec, Therwil, Switzerland) (study group), or recruited to the control group if a TCpCO_2_ monitor was not available. **Results**: A total of 132 ELBW infants participated in the study. The size of the study group (106 infants) and the control group (26 infants) differed because monitor availability increased during the study period reflecting change in standard of care. The groups had comparable gestational age and baseline characteristics. No difference was found in the rate of IVH/PVL in the study vs. control groups (10% vs. 4%; *p* = 0.7, respectively), or in the combined outcome of PVL/IVH and death (16% vs. 15%; *p* = 1.0, respectively). **Conclusions**: This study demonstrates the challenges in conducting a prospective controlled trial in a rapidly evolving medical field. While the study began with a clear equipoise, this balance shifted as the care team gained more experience with TCpCO_2_ monitoring among the study population, despite the absence of new clinical evidence to justify such a shift. Consequently, the small control group limited our ability to draw definitive conclusions regarding the study’s objective. However, our findings may increase awareness of continuous non-invasive pCO_2_ monitoring in extremely premature infants.

## 1. Introduction

Maintaining very low birth weight (VLBW) premature infants within a predefined CO_2_ range is essential, as both hypocarbia and hypercarbia are associated with neurological and respiratory morbidities [1,2,3,4]. Hypocarbia has been linked to an increased risk of bronchopulmonary dysplasia (BPD) [3], intraventricular hemorrhage (IVH) [1,3], and periventricular leukomalacia (PVL) [2,3] in premature infants, while hypercarbia has been associated with an elevated risk of IVH in this population [1,5,6,7]. Fluctuations in PaCO_2_ have also been correlated with severe IVH [1,6].

To mitigate these risks, CO_2_ levels are frequently measured in blood samples. However, due to rapid changes in respiratory status of these extremely premature infants, both high and low CO_2_ levels may go undetected [8,9]. Non-invasive, continuous pCO_2_ monitoring options in the neonatal intensive care unit (NICU) include end-tidal CO_2_ (EtCO_2_) monitoring and transcutaneous pCO_2_ (TCpCO_2_) monitoring. Each monitoring method possesses distinct advantages and disadvantages [7,10,11]. In a previous publication we demonstrated that infants continuously monitored by EtCO_2_ spent more time within a predefined safe pCO_2_ range (30–60 mmHg) during conventional ventilation [9]. The monitored group exhibited lower rates of IVH and PVL. However, the number of VLBW infants in the study with abnormal US findings was relatively small. Thus, we were cautious in concluding that monitoring reduced the rate of neurological morbidities.

TCpCO_2_ monitoring is being increasingly adopted in the NICU [12,13] and offers continuous non-invasive monitoring of pCO_2_ levels in premature infants, regardless of their respiratory support needs, type of ventilation (invasive or non-invasive), or mode of ventilation (conventional mechanical ventilation [CMV] or high-frequency oscillatory ventilation [HFOV]). Good correlation and agreement between TCpCO_2_ and blood gas pCO_2_ measurements have been demonstrated in most studies of premature infants, although the limit of agreement was mostly wide [14,15,16,17,18]. However, despite the increased use of this monitoring method in the NICU, its impact on the clinical outcomes of VLBW premature infants has not yet been determined in a large, prospective trial. This uncertainty surrounding the impact of TCpCO_2_ monitoring on ELBW premature infants during the first week of life reflects the clinical equipoise at the beginning of the study. While clinical and biophysical logic suggests that continuous pCO_2_ monitoring in this vulnerable population could lead to improved CO_2_ control and reduction in neurological morbidities, this assumption must be validated in a large study. Potential harms, arising from technical issues or false alarms that could confuse the care team, must also be considered. We hypothesized that continuous pCO_2_ monitoring could reduce the rate of short-term neurological complications by lowering exposure to hypocarbia and hypercarbia during the most critical time period in the most vulnerable premature infants. Thus, the aim of our study was to evaluate whether continuous non-invasive pCO_2_ monitoring, using a TCpCO_2_ monitor during the first week of life, would decrease the rate of high-grade IVH or PVL among extremely low birth weight (ELBW) premature infants and the combined outcome of IVH or PVL and death.

## 2. Materials and Methods

This was a prospective, observational, multicenter study conducted in seven NICUs in Israel (ClinicalTrial.gov. ID NCT03477708). This study was not conducted as a randomized controlled trial (RCT) due to ethical concerns raised by the research ethics boards. They argued that it would be unethical to prevent monitoring of an infant simply because of randomization, when a monitor was available and not in use. As a result, we used “ethical randomization” based on the availability of monitors. The study was approved by the research ethics boards of all centers participating in the study. Written informed consent was obtained from parents of all infants prior to study entry.

Our primary outcome was high-grade IVH or PVL before hospital discharge. Secondary outcomes included death, BPD, retinopathy of prematurity (ROP), necrotizing enterocolitis (NEC), sepsis, hematocrit (HCT) at 7 days and at discharge, and need for red-blood-cell transfusion.

### 2.1. Study Population

All premature infants <1000 g admitted to the participating NICUs during the study period and needing respiratory support during the first day of life were eligible to be included in the study. Infants with severe congenital malformation, birth asphyxia (defined as arterial cord pH < 7.1 and an abnormal neurological exam by 6 h of age), known intraventricular hemorrhage stage III–IV in the first 24 h of life (likely attributed to perinatal events during periods when TCpCO_2_ is not monitored), or for whom active treatment was not initiated, were excluded from the study.

As previously discussed, for ethical reasons, allocation was based on monitor availability. Each infant <1000 g admitted to a participating NICU and who was monitored by TCpCO_2_ monitor (Sentec, Therwil, Switzerland) from the first day of life for at least one week was included in the study group. If a TCpCO_2_ monitor was not available upon NICU admission, the infant was recruited to the control group. Respiratory support included invasive (CMV and HFOV) and non-invasive support including nasal intermittent positive pressure ventilation (NIPPV), continuous positive airway pressure (CPAP), and heated humidified high flow nasal cannula (HHHNC). Routine care was provided to all infants regardless of their allocation.

### 2.2. Study Design

TCpCO_2_ monitoring was started during the first 12 h of life. Probe placement was in predefined areas and sensor temperature was set to 41 °C in accordance with the manufacturer’s instructions [16]. Calibration of the TCpCO_2_ was automatically performed every 4 h and following any reposition of the probe. Sensor membranes were changed every 28 days, or sooner in case of any visible damage, such as small tears at the periphery of the membrane, or repeated calibration errors. Skin fixation adhesives and contact gel were used in accordance with manufacturer’s guidelines.

Head US was performed on day 3 of life, day 10 of life, during the 4th week of life, and at term-equivalent age, as well as on any other occasion at the discretion of the medical team. IVH was classified according to the criteria of Papile et al. [19], BPD was defined as the need for supplemental O_2_ or positive pressure ventilation support at 36 weeks corrected age [20], ROP grading and treatment were defined by the guidelines of the Early Treatment for Retinopathy of Prematurity Cooperative Trial [21], and NEC was defined according to Bell criteria [22].

No predefined TCpCO_2_ values were established for blood gas sampling or changes in respiratory support as this was an observational study. Blood samples for blood gas analysis were taken at the discretion of the care team, as were changes in respiratory support. Target blood gas pCO_2_ levels were maintained according to our routine practice, at between 35 and 60 mmHg.

To address the difference in group size, we conducted matching between the study and control groups. Matching for ELBW infants was based on the study center and date of birth. For each infant in the control group, we identified two infants from the study group who were born before and after.

### 2.3. Statistical Analysis

Sample-size calculation was carried out based on data from the Israeli Neonatal Network annual report [23]. For a decrease in the combined outcome of severe IVH and PVL from 30% to 15% (a decrease of 50%), the sample calculation with 80% power and alpha of 0.05 using two-sided *t*-test resulted in a sample size of 121 infants per group.

Descriptive statistics in terms of mean, standard deviation, percentage, and ranges were calculated as needed. Normal distribution of the continues parameters were tested by Kolmogorov–Smirnov test and data are presented as mean ± standard deviation (SD) for normally distributed variables, or median with interquartile range (IQR) for variables with non-parametric distribution. We used the *t*-test or Mann–Whitney U test to compare the study and control groups. For categorical parameters, we used Fisher’s exact test or Pearson’s chi-squared test. *p* < 0.05 was considered as significant. SPSS version 28 used for all statistical analyses (IBM SPSS, Chicago, IL, USA).

## 3. Results

**Figure 1:**
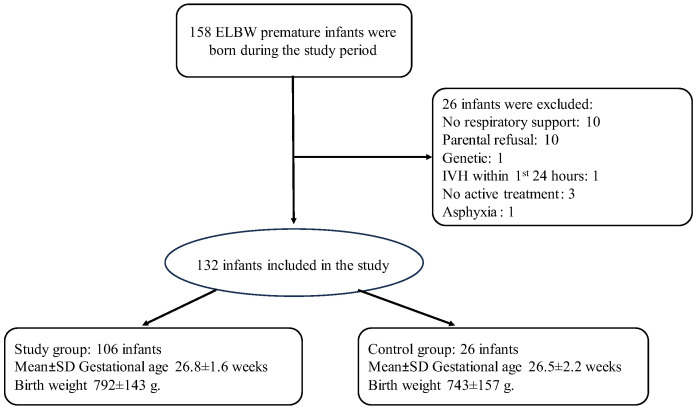
Recruitment chart.

A total of 158 ELBW premature infants were born during the study period between March 2018 and September 2021 (Figure 1).

We recruited 132 ELBW infants to the study: 106 infants in the study group and 26 infants in the control group. Group size differed due to increased monitor availability in the participating NICUs, reflecting a change in the standard of care in Israel during the study period. To address the differences in group sizes, we conducted two separate statistical analyses: one for the full sample of infants in each group and another for matched groups. In the latter analysis, two infants from the study group (n = 52 infants) were matched with each infant from the control group (n = 26 infants) born in the same center at the same time period.

Table 1 shows the baseline characteristics of the infants. All comparisons between the study, control, and matched groups were not significant, with the exception of cord pH, which reached statistical (*p* = 0.04), though not clinical, significance. Four infants required resuscitation in the delivery room, including chest compression—three from the study group and one from the control group. None of these infants died or experienced IVH/PVL (Table 1).

Table 2 shows the clinical primary and secondary outcomes. No difference was found in the rate of high-grade IVH or PVL in the study and matched groups vs. controls (10% and 6% vs. 4%, *p* = 0.7 and 1.0, respectively), or in the combined outcome of high-grade IVH, PVL, or death (16% and 15% vs. 15%, *p* = 1.0 and 1.0, respectively). The secondary outcomes including IVH of any grade, death, length of stay, BPD, sepsis, PDA, and HCT level were also similar (Table 2). ROP was significantly higher in the control group compared with the matched study group, but not when compared to the study group. Of all infants with high-grade IVH, two infants were diagnosed with IVH on day 28 HUS, following normal HUS on days 3 and 10. We did not observe any burns or skin breakdowns among the participating infants.

Among infants who survived the first week of life, the number of blood samples per infant tended to be higher in the study vs. control groups but was comparable between the matched and control groups (Table 3).

Data on the agreement and correlation between TCpCO_2_ and blood samples in our cohort were recently published [18]. The average number of samples per infant that were found in the hypocarbic range during the first week of life (pCO_2_ < 35 mmHg) and in the hypercarbic range (pCO_2_ > 60 mmHg) in the study vs. control groups is presented in Table 3.

## 4. Discussion

Our study was initially designed as an RCT to evaluate the impact of TCpCO_2_ monitoring on neurological outcome among ELBW premature infants. However, despite careful planning, we encountered significant challenges due to ethical constraints and a shift in equipoise during the recruitment process, despite the absence of new clinical evidence to justify such a shift. This led to a small control group and limited our ability to achieve the study’s original objectives. Despite this limitation, our findings may increase awareness of continuous non-invasive pCO_2_ monitoring in extremely premature infants and underscores the challenges of conducting clinical research in this vulnerable population within a rapidly evolving field.

Advances in neonatal care have led to increased survival rates among extremely premature infants. Nevertheless, the incidence of high-grade IVH and PVL has remained constant [24]. Although the clinical equipoise regarding our study aim was initially clear, recent literature [12,13,25], and the increased awareness of TCpCO_2_ monitoring among bedside teams, affected our study process. European surveys in Germany, Austria, Switzerland, and the Netherlands reported that most NICUs (at least 76%) use transcutaneous blood gas monitoring [13,25]. In Japan transcutaneous monitoring was used in 85% of institutions [12] despite the fact that most participants (93%) felt the accuracy was moderate. We lack data regarding beliefs of care teams in Israel, but the demand to incorporate additional TCpCO_2_ monitors to facilitate continuous pCO_2_ monitoring of as many ELBW infants as possible demonstrates that care teams find reassurance in using TCpCO_2_ for continuous control of pCO_2_ levels. The increased use of TCpCO_2_ monitoring in our study, derived solely by the care team’s growing familiarity with this technique, and not by new clinical evidence supporting its advantages in this population, led to a shift in equipoise and created tension between the need for a prospective controlled trial and the ethical constraints involved.

In his pioneering work in 1974, Charles Fried [26] defined equipoise as a genuine uncertainty regarding the relative merits of different arms in a clinical trial, asserting that it should be established as an ethical precondition for research, regardless of potential third-party benefits. A decade later, Freedman highlighted the problem with the requirement that the investigators maintain no “treatment preference” throughout the course of a trial, as this poses a significant obstacle to completing controlled trials and may lead to their termination because of the failure to enroll enough patients [27]. Our study exemplifies this exact problem. Starting this study, we had a clear clinical equipoise. However, this equipoise was shifted once recruitment began, and the bedside teams started using TCpCO_2_ monitors in this population of tiny premature infants and developed “treatment preference”. Given these recruitment challenges and the physiological rationale for continuous CO_2_ monitoring in this population, our study may suggest that conducting a larger RCT to address this research question would be impractical.

The small control group mandates caution while interpreting the results of our study. Our study revealed no significant difference in the rates of high-grade IVH or PVL in both study and matched groups compared to controls or in the combined outcome of high-grade IVH, PVL, and death. Secondary outcomes, including BPD, also showed no significant differences. Our study may suggest a lower rate of ROP among the monitored group, possibly due to the avoidance of elevated CO_2_ levels [28,29]. Yet, our study lacked sufficient power to fully address these aspects. A retrospective study by Mukhopadhyay et al., which included more mature infants (approximately 28 weeks GA, and >1000 g), comparing periods before and after the implementation of TCpCO_2_ monitoring, reported similar findings, of no significant effect on clinical outcomes, including IVH and PVL, with the use of TCpCO_2_ monitoring [15].

In our study, we observed lower rates of both IVH and PVL in both the study and control groups when compared with the anticipated rate derived from the Israeli Neonatal Network annual report [23] and existing literature [24]. We cannot attribute the reduction in short-term neurological outcomes to TCpCO_2_ monitoring during this high-risk period; rather, we propose heightened vigilance across both study and control groups as a potential contributing factor.

In a separate publication using the same cohort [18], we observed a moderate correlation between TCpCO_2_ and bgCO_2_ measurements and trends. TCpCO_2_ measurements demonstrated good agreement (bias < 5 mmHg) with bgCO_2_ regardless of day of life, ventilation mode (invasive/non-invasive), or sampling method (arterial/capillary/venous), but with wide limits of agreement (LoA) (lower LoA, upper LoA—11.8, 17.6). Therefore, we concluded that to assess pCO_2_ levels and trends in individual patients, TCpCO_2_ should be used as a complementary tool to blood gas sampling. These findings were found among the tiniest infants in the NICU and in the humid environment in the incubator during the first week of life. Our results align with those of other studies using TCpCO_2_ monitoring in premature infants [14,15,16,17,30].

In our study we found no difference in the number of samples with pCO_2_ measurements outside the target pCO_2_ range. These findings are similar to those of Mukhopadhyay et al. [15]. This may indicate that clinicians have become more adept at maintaining pCO_2_ levels within the target range, potentially reducing the frequency of extreme deviations associated with IVH/PVL. This could explain the lack of observed difference in IVH/PVL between the groups. However, this does not necessarily imply that infants spent an equivalent amount of time in safe or unsafe pCO_2_ zones, as we do not have records to quantify the area under the pCO_2_ curve in order to address this question. Similar findings were reported by us previously [9].

TCpCO_2_ monitoring has the potential to reduce repeated blood gas draws, thereby minimizing iatrogenic blood losses. This might be particularly significant during the first week of life when the infants are unstable, and especially in the presence of arterial lines, which makes blood sampling easier. However, not fully trusting the method, or false alarms resulting from high limits of agreement or technical problems, may inadvertently lead to increased blood draws. Mukhopadhyay et al. found that TCpCO_2_ monitors were associated with decreased blood gas sampling throughout the entire NICU stay [15]. In our study, the HCT tended to be lower at the end of the first week in the control group but the need for blood transfusion was comparable between groups. Multiple factors can influence blood draws and transfusions in the first week of life in ELBW infants. To draw definitive conclusions on this issue, a multivariate analysis should be conducted in a study with sufficient power.

The main limitation of our study was the recruitment process, which prevented us from reaching the target sample size for the control group. By the time we completed recruitment to the study group, we had only recruited about a quarter of the control group. We decided to cease recruitment as continuing recruitment would have further increased the imbalance between the study and control groups. Attempting to achieve full recruitment to the control group, or even only doubling its size, was impractical. Due to the imbalance between the control and study groups, we considered using a historical control. However, the implementation of advanced care practices and the adoption of a new bundle of care for IVH prevention in Israel made the use of an historical control group unfeasible. Instead, we used a matched control group, as described in our methods. This led to a reduction in the number of participants assessed in the study group. We cannot rule out a potential beta error—an undetected clinical effect of TCpCO_2_. In addition, the incidence of IVH and PVL was lower than expected and used in the sample-size calculation, further increasing the risk of beta error.

The strength of our study was in demonstrating the feasibility and safety of this monitoring method even in the smallest premature infants during their first days of life, despite their fragile skin and the humid environment in the incubators. Moreover, the agreement and correlation observed in the current cohort [18] support the use of this method, provided that its associated limitations are well understood by medical teams. Our study was intended to be the first prospective study investigating the impact of continuous pCO_2_ monitoring in extremely premature infants during the most critical time period. This important clinical question remains unsolved.

In conclusion, this study demonstrates the challenges of conducting a prospective controlled trial in a rapidly evolving medical field. The study began with a clear equipoise that shifted as the care team became more familiar with TCpCO_2_ monitoring among the study population, despite the absence of new clinical evidence to justify a true shift in equipoise. This resulted in a small control group, not allowing us to reach a conclusion on the study objective. Nonetheless, our study increases awareness of continuous non-invasive pCO_2_ monitoring in extremely premature infants.

## Figures and Tables

**Table 1:** Infants’ baseline characteristics.

	Study N = 106	Control N = 26	Matching from Study Group N = 52
GA, mean ± SD	26.8 ± 1.6	26.5 ± 2.2	26.9 ± 1.8
BW, mean ± SD	792 ± 143	743 ± 157	762 ± 158
SGA, n (%)	9 (8)	3 (11)	9 (17)
Male, n (%)	46 (44)	11 (42)	21 (41)
Twins, n (%)	28 (27)	7 (27)	13 (26)
Vaginal delivery, n (%)	24 (23)	6 (23)	13 (26)
Prenatal steroids, n (%)	80 (80)	20 (77)	41 (85)
Chorioamnionitis, n (%)	11 (11)	2 (8)	3 (6)
Preeclampsia, n (%)	27 (27)	5 (20)	13 (27)
Apgar 5 min, mean ± SD	8 ± 2	8 ± 2	8 ± 2
Intubation at birth, n (%)	43 (42)	7 (27)	20 (41)
Umbilical cord pH, mean ± SD	7.27 ± 0.1	7.21 ± 0.1 *	7.27 ± 0.1
Umbilical cord BE, mean ± SD	−5 ± 4	−7 ± 3	−5 ± 4

* *p* < 0.05. GA, gestational age; BW, birth weight; SGA, small for gestational age.

**Table 2:** Primary and secondary outcomes.

	Study N = 106	Control N = 26	Matching from Study Group N = 52	*p* Value Study vs. Control	*p* Value Matched vs. Control
IVH grade >2 or PVL	11 (10)	1 (4)	3 (6)	0.7	1.0
IVH grade >2 or PVL or death	17 (16)	4 (15)	8 (15)	1.0	1.0
Any IVH	29 (28)	7 (29)	15 (29)	1.0	1.0
Death	10 (9)	3 (11)	7 (13)	0.72	1.0
Length of stay (days)	91 ± 39	93 ± 41	91 ± 49	0.84	0.88
BPD	41 (44)	6 (27)	19 (44)	0.23	0.28
Culture-proven sepsis	28 (28)	5 (22)	12 (24)	0.61	1.0
ROP needing treatment	6 (6)	4 (17)	1 (2)	1.0	**0.04**
PDA needing treatment	38 (36)	8 (33)	19 (37)	0.82	0.80
HCT day 8 (%)	39 ± 6	36 ± 6	39 ± 6	0.06	0.08
Packed-cell transfusions during first week	52 (55)	16 (73)	28 (56)	0.15	0.20

Data are presented as n (%) or mean ± SD. IVH, intraventricular hemorrhage; PVL, periventricular leukomalacia; BPD, bronchopulmonary dysplasia; ROP, retinopathy of prematurity; PDA, patent ductus arteriosus; HCT, hematocrit.

**Table 3:** Blood sampling among the different groups and in different ranges.

	Study N = 106	Control N = 26	Matching from Study Group N = 52	*p* Value Study vs. Control	*p* Value Matched vs. Control
No. of samples/infant during the first week of life	21.5 ± 7.6	18.6 ± 7.6	20.3 ± 7.7	0.053	0.15
No. of samples/infant in the hypocarbic range during the first week of life	2.1 ± 2.7	1.3 ± 1.4	2.2 ± 3.0	0.13	0.09
No. of samples/infant in the hypercarbic range during the first week of life	1.5 ± 2.0	2.0 ± 3.1	1.3 ± 1.9	0.30	0.19

Data are presented as mean ± SD. Hypocarbic range, pCO_2_ < 35 mmHg. Hypercarbic range, pCO_2_ > 60 mmHg.

## Data Availability

Data are available upon reasonable request from the corresponding author.
